# Supplementary material for: Latent developmental and evolutionary shapes embedded within the grapevine leaf
Source: New Phytol. 2015 Nov 18;210(1):343–55. doi: 10.1111/nph.13754 (PMC5063178; doi:10.1111/nph.13754)

New Phytologist

***New Phytologist* Supporting Information Figs S1–S4**

Article title: Latent developmental and evolutionary shapes embedded within the grapevine leaf

Authors: Daniel H. Chitwood, Laura L. Klein, Regan O'Hanlon, Steven Chacko, Matthew Greg, Cassandra Kitchen, Allison J. Miller and Jason P. Londo

Article acceptance date: 13 October 2015

The following Supporting Information is available for this article:

Fig. S1 Numbers of species, vines, leaves, and shoot positions sampled.

Fig. S2 Developmental stage and leaf number are partially confounded.

Fig. S3 Projection of species with ≥5 vines onto the morphospace.

Fig. S4 Comparison of leaf shape changes due to developmental stage (Sn) and leaf number (Ln) for different species.

Table S1 Procrustes-aligned coordinates (separate Excel file)

Table S2 Correlation between traits (separate Excel file)

Fig. S1 Numbers of species, vines, leaves, and shoot positions sampled. (a) Leaves per species, arranged in descending order. (b) Vines sampled per species. For some analyses in the paper, only those *Vitis* species with >5 vines were analyzed (indicated by red line). (c) The average number of leaves sampled per vine for each species. (d) Number of leaves represented at each developmental stage. For some analyses, only developmental stage ≤S10 were analyzed (indicated by red line). (e) Number of leaves represented at each leaf number position. For some analyses, only leaf numbers ≤L10 were analyzed (indicated by red line).

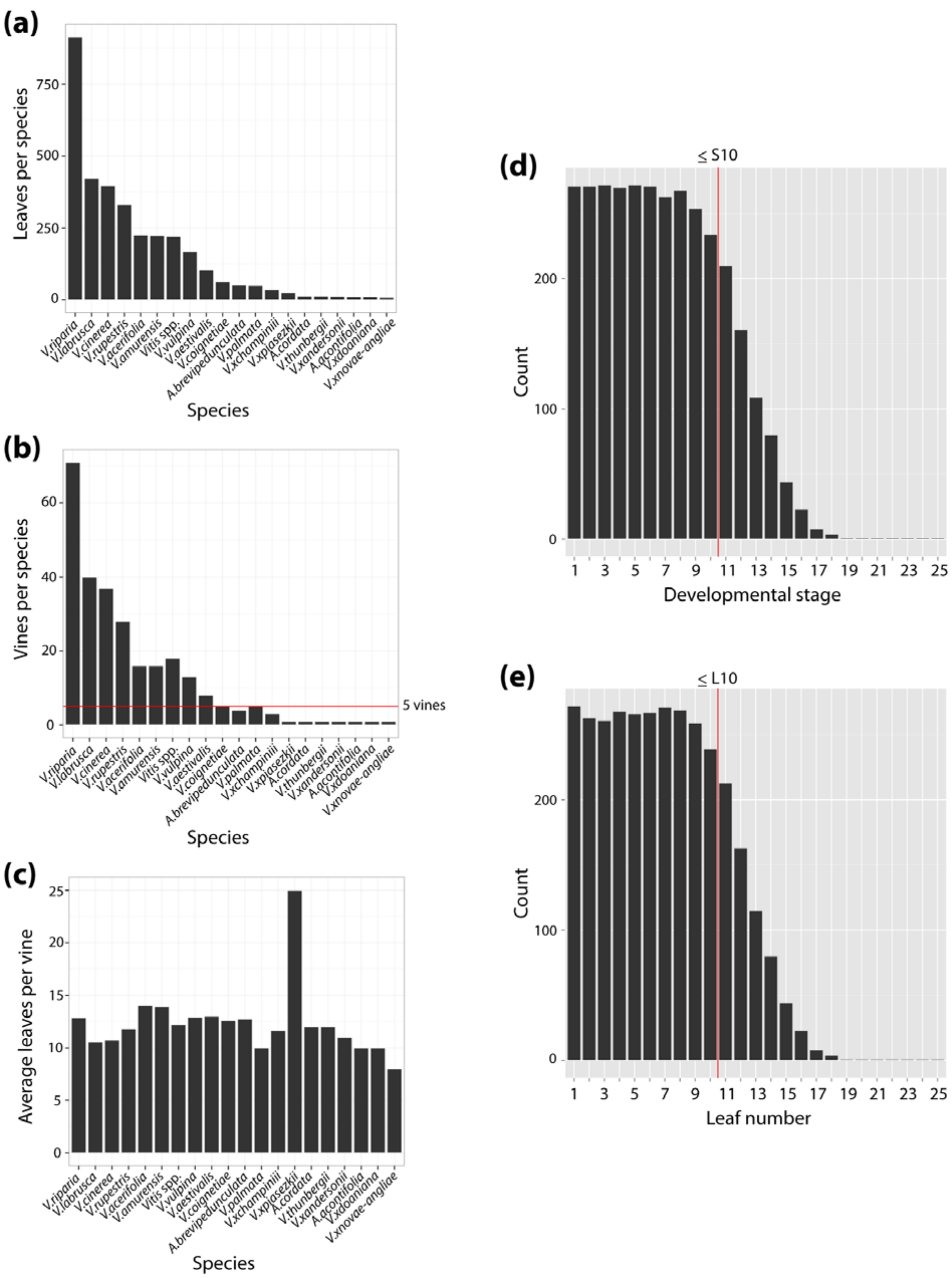
(a)
Leaves per species
750
500
250
0
Species
(b)
Vines per species
60
40
20
0
5 vines
Species
(c)
Average leaves per vine
25
20
15
10
5
0
Species
V.riparia
V.labrusca
V.cinerea
V.rupestris
V.acerifolia
V.amurensis
Vitis spp.
V.vulpina
V.aestivalis
V.coignetiae
A.brevipedunculata
V.palmata
V.xchampinii
V.xpiasezkii
A.cordata
V.thunbergii
V.xandersonii
A.acontifolia
V.xdoaniana
V.xnovae-angliae
(d)
≤ S10
Count
200
100
0
1 3 5 7 9 11 13 15 17 19 21 23 25
Developmental stage
(e)
≤ L10
Count
200
100
0
1 3 5 7 9 11 13 15 17 19 21 23 25
Leaf number

Fig. S2 Developmental stage and leaf number are partially confounded. (a) Leaves (shown in Fig. 1) from different species aligned by developmental stage (Sn), beginning at the shoot tip. Because of variability in shoot length, the developmental stage of each node corresponds to different leaf numbers between shoots. Developmental stage is similar to plastochron number (Pn), a classic indexing method used in developmental studies of leaves. (b) Similar to (a) except that leaves are aligned by leaf number (Ln), beginning at the shoot base. Because of variability in shoot length, the leaf number of each node corresponds to different developmental stages between shoots. Because developmental stage and leaf number are oppositely numbered, beginning with shoot tip and base, respectively, their effects on leaf morphology are generally opposite of each other. However, because of variability in shoot length, specific effects for each can be discerned to a limited degree. *V. rip.*, *V. riparia*; *V. aes.*, *V. aestivalis*; *V. thu.*, *V. thunbergii*; *V. lab.*, *V. labrusca.*

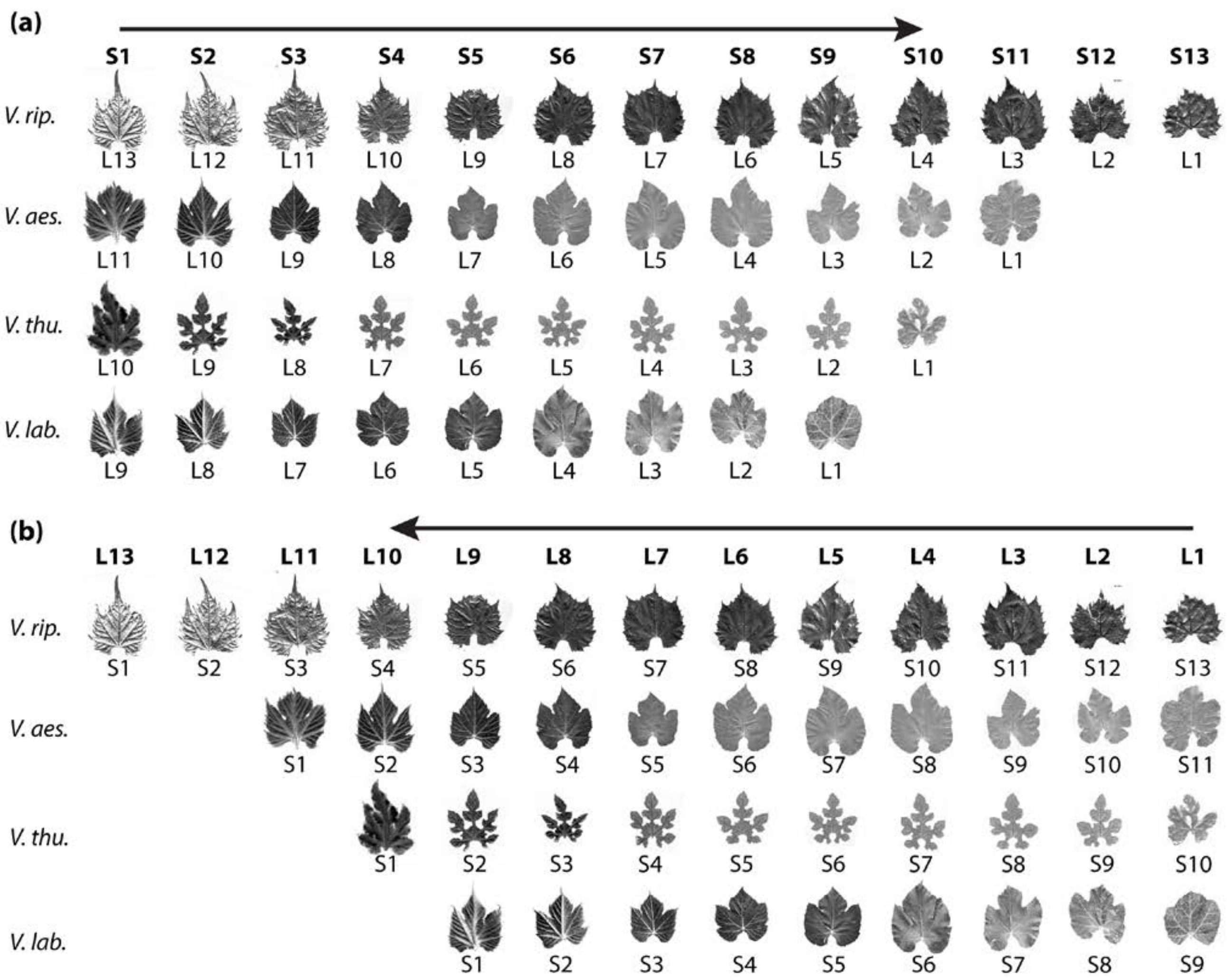
(a)
S1 S2 S3 S4 S5 S6 S7 S8 S9 S10 S11 S12 S13
V. rip.
L13 L12 L11 L10 L9 L8 L7 L6 L5 L4 L3 L2 L1
V. aes.
L11 L10 L9 L8 L7 L6 L5 L4 L3 L2 L1
V. thu.
L10 L9 L8 L7 L6 L5 L4 L3 L2 L1
V. lab.
L9 L8 L7 L6 L5 L4 L3 L2 L1
(b)
L13 L12 L11 L10 L9 L8 L7 L6 L5 L4 L3 L2 L1
V. rip.
S1 S2 S3 S4 S5 S6 S7 S8 S9 S10 S11 S12 S13
V. aes.
S1 S2 S3 S4 S5 S6 S7 S8 S9 S10 S11
V. thu.
S1 S2 S3 S4 S5 S6 S7 S8 S9 S10
V. lab.
S1 S2 S3 S4 S5 S6 S7 S8 S9

New Phytologist

Fig. S3 Projection of species with ≥5 vines onto the morphospace. (a) Species with ≥5 vines (indicated by color) projected onto the morphospace. Leaves from other species are indicated with gray points. Visualized data includes all shoot positions. (b) Same data as in (a) with 95% confidence ellipses for the highlighted species, indicated by color.

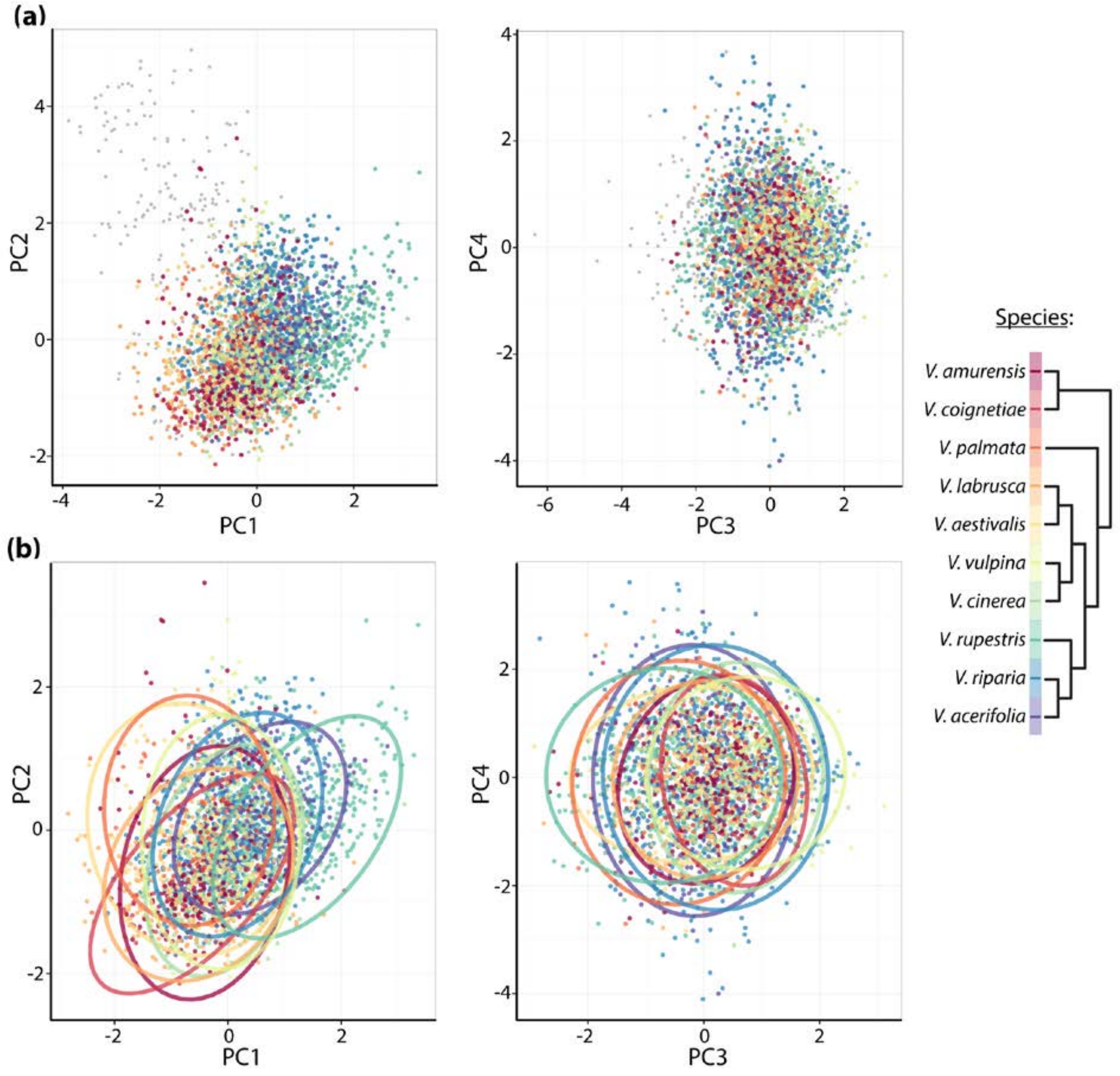

New Phytologist

Fig. S4 Comparison of leaf shape changes due to developmental stage (Sn) and leaf number (Ln) for different species. Each graph compares changes in a principal component (PC1, PC2, or PC3) against developmental stage (Sn, orange) and leaf number (Ln, blue) along the *x*-axis ('Shoot position') for each *Vitis* spp. for which ≥5 vines were sampled as a locally weighted scatterplot smoothing (LOWESS) curve.

New Phytologist

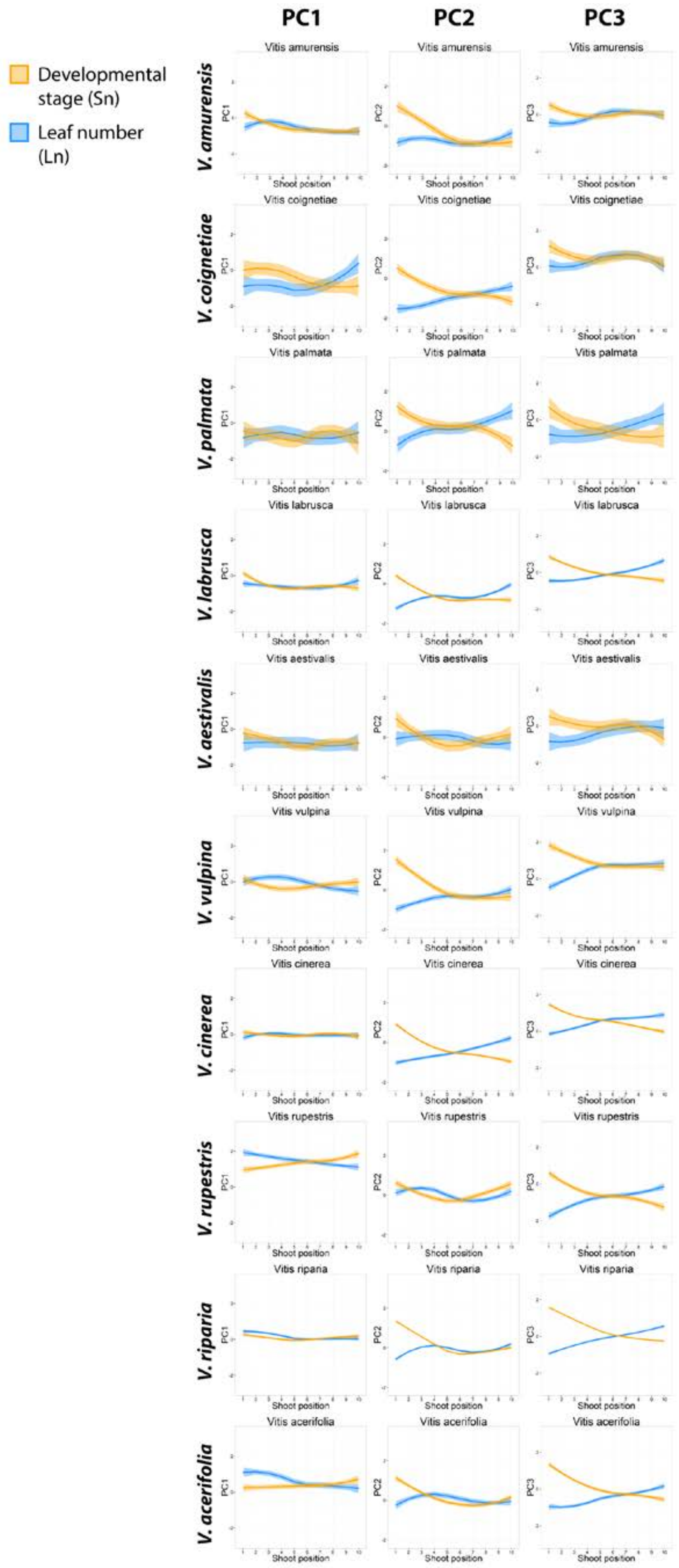

Supplement: Supplementary file 1 — Fig. S1 Numbers of species, vines, leaves and shoot positions sampled. Fig. S2 Developmental stage and leaf number are partially confounded. Fig. S3 Projection of species with five vines or more onto the morphospace. Fig. S4 Comparison of leaf shape changes caused by developmental stage (Sn) and leaf number (Ln) for different species. [file NPH-210-343-s001.pdf]
